# Targeting ubiquitination machinery in cystic fibrosis: Where do we stand?

**DOI:** 10.1007/s00018-024-05295-z

**Published:** 2024-06-18

**Authors:** Tsukasa Okiyoneda, Christian Borgo, Valentina Bosello Travain, Nicoletta Pedemonte, Mauro Salvi

**Affiliations:** 1 Department of Biomedical Sciences, School of Biological and Environmental Sciences, Kwansei Gakuin University, Hyogo, 669-1330 Japan; 2 Department of Biomedical Sciences, University of Padova, 35131 Padova, Italy; 3 Department of Medicine, University of Padova, 35128 Padova, Italy; 4 Department of Molecular Medicine, University of Padova, 35131 Padova, Italy; 5 UOC Genetica Medica, IRCCS Istituto Giannina Gaslini, Via Gerolamo Gaslini 5, 16147 Genoa, Italy

**Keywords:** Protein misfolding, Small molecules, CFTR therapy, Protein stability, Post-translational modifications

## Abstract

Cystic Fibrosis (CF) is a genetic disease caused by mutations in *CFTR* gene expressing the anion selective channel CFTR located at the plasma membrane of different epithelial cells. The most commonly investigated variant causing CF is F508del. This mutation leads to structural defects in the CFTR protein, which are recognized by the endoplasmic reticulum (ER) quality control system. As a result, the protein is retained in the ER and degraded via the ubiquitin–proteasome pathway. Although blocking ubiquitination to stabilize the CFTR protein has long been considered a potential pharmacological approach in CF, progress in this area has been relatively slow. Currently, no compounds targeting this pathway have entered clinical trials for CF. On the other hand, the emergence of Orkambi initially, and notably the subsequent introduction of Trikafta/Kaftrio, have demonstrated the effectiveness of molecular chaperone-based therapies for patients carrying the F508del variant and even showed efficacy against other variants. These treatments directly target the CFTR variant protein without interfering with cell signaling pathways. This review discusses the limits and potential future of targeting protein ubiquitination in CF.

## Introduction

Cystic fibrosis (CF) is caused by variants in the gene encoding the cystic fibrosis transmembrane conductance regulator (CFTR), an anion selective channel present in epithelial tissues [1]. Over 2000 variants in the *CFTR* gene have been reported, of which about 700 are considered pathogenic [2]. Pathological variants have the potential to cause a variety of issues within proteins. These include absence or reduced protein expression (class I variants), defects in processes such as protein trafficking/maturation (class II variants), gating mechanisms (class III variants), conduction properties (class IV variants), protein amount (class V variants) and compromised protein stability at the plasma membrane (PM) (class VI variants) [1]. This led to the classification of a variant in different classes based on the type of defect [3]. However, it is important to note that a single variant has the potential to simultaneously influence various properties of a protein, which can complicate its straightforward categorization into a single class. The most common CF variant, F508del, has been attributed to a class II variant, which causes defective trafficking and rapid degradation via the ubiquitin-proteasomal pathway due to protein folding defects at the endoplasmic reticulum (ER) [4–6]. However, F508del-CFTR also presents defects in gating (typical of class III variants) and instability at the PM (associated with variants of class VI) when it reaches the cell surface upon low-temperature incubation and/or corrector molecules [3].

Research on the functional recovery of CF variants has obtained several successes over the years. The first effective drug, VX-770 (ivacaftor (IVA); trade name Kalydeco^®^), a CFTR potentiator that increases CFTR channel activity at the cell surface, was developed following high-throughput pharmaceutical screening to treat G551D variant, the prototype of gating variants [7, 8]. IVA represented a breakthrough in CF research [9]. Although the use of this drug was extended to different gating and conductance variants (Kalydeco^®^ is approved by FDA for people with CF ages 1 month and older having one of 97 specific variants, https://www.cff.org/news/2023-05/fda-approves-kalydeco-infants), they only account as a single molecule treatment for a limited percentage of patients (the frequency of the G551D worldwide is estimated to be between 1 and 5%. [10]). Nevertheless, the discovery was significant because it demonstrated the potential to treat genetic diseases, such as CF, by correcting their molecular defects with small molecules.

In the following years, there was extensive research focused on finding a treatment for the prevalent CFTR variant, F508del, characterized by folding and trafficking defects. Great efforts were employed for the identification of correctors, i.e. drug-like small molecules able to improve the maturation and trafficking defects, increasing its cell-surface levels. Numerous compounds behaving as correctors were identified by high-throughput screening [11], albeit with generally low efficacy. Two different mechanisms of action were initially postulated for compounds acting as correctors [12]. Indeed, these compounds may exert their activity by directly binding to F508del-CFTR, thus improving the defective folding of the mutant protein. These kinds of correctors are referred to as pharmacological chaperones, to recall the molecular chaperones (such as Hsp90) that intervene to promote protein folding [12]. Some of the identified correctors were however more likely to act by an alternative mechanism of action, i.e. by modulating (directly or indirectly) specific proteins or pathways that are involved in or may have an impact on CFTR protein folding or degradation. These correctors are referred to as proteostasis regulators [12].

VX-809 (lumacaftor (LUM)) emerged as one of the first most promising compounds being able to stabilize the F508del-CFTR immature form and showing the greatest rescue efficacy compared to previously identified molecules [13]. The mechanism of action has recently been disclosed. The cryo-electron microscopy structures of wild-type (WT) and F508del-CFTR in complex with LUM show that the molecule inserts into a hydrophobic pocket in transmembrane domain 1 (TMD1) increasing its stability [14, 15]. In 2015, FDA and EMA approved the co-treatment with LUM and the potentiator IVA (Orkambi^®^, Vertex Pharmaceuticals) for F508del-homozygous patients aged ≥ 12 years [16]. Nevertheless, the use of this combination of two drugs resulted in modest clinical improvements in patients [17–19].

The turning point came with the emergence of new correctors and the combination of two of these, VX-661 (tezacaftor (TEZ)) and VX-445 (elexacaftor (ELX)). This marked a pivotal moment in CF research, as this corrector combination plus the potentiator IVA (Trikafta^®^, Kaftrio^®^ in Europe, Vertex Pharmaceuticals, Cambridge, MA) displayed remarkable outcomes, far surpassing Orkambi^®^ effects, both in vitro and, more importantly, in clinical studies [20]. It has now become the forefront of current pharmacological treatment for CF patients who have at least one F508del variant in the *CFTR* gene and it has been extended to several other misfolded variants (to date, Trikafta has been approved for 178 mutations, see https://www.trikafta.com/sites/default/files/patient-brochure.pdf) and the possibility of expanding its use is still under investigation [21, 22]. TEZ is a structural analog of LUM with the same mechanism of action [14] (both molecules are classified as type I correctors). ELX stabilizes TM 10–11, thereby strengthening the TMD/NBD1 interface [15] (type III corrector). The combination of ELX and TEZ targeting different domains of the protein acts synergistically to improve CFTR folding [15]. Moreover, ELX binding has a dual function, improving CFTR folding as well as ion conductance [23]. Notably, other correctors acting as pharmacological chaperones have recently been identified and are under evaluation in clinical trials [24]. Therefore, currently, all available or in-development pharmacological treatments are designed to directly target the defective channel itself without any immediate impact on cell signaling.

According to the different mechanisms of action, correctors acting as pharmacological chaperones may exert their effects also on other proteins structurally related to CFTR, namely other ABC proteins, as demonstrated in the case of the phosphatidylcholine transporter ABCB4, whose misfolded variants can be rescued by C18, a structural analog of LUM [25]. On the other hand, proteostasis regulators may be utilized to stabilize mutant proteins other than CFTR, including those not structurally-related to CFTR [26]. However, their activity is markedly influenced by cell background since it depends on the expression level and biological role of their targets in the different cell models [12, 27]. This also explains the identification of several classes of putative CFTR correctors (likely acting as proteostasis regulators) that displayed a cell type-specific activity [27]. Probably for this reason, in the last years the use of disease-relevant cell models has become more common in the path towards the identification of novel drugs to overcome the CF basic defect.

Over the years, alternative approaches have been pursued to address variants with folding anomalies that are responsible for trafficking defects and protein degradation. This approach involves intervention not directly at the channel level, but rather within cell signaling pathways. This strategy,  focusing on proteostasis regulators, targets components of the CFTR regulome, such as chaperones, cochaperones, kinases, or ubiquitin ligases that affect the synthesis, folding, stability, and trafficking to the PM of the mutant channel [28].

**Fig. 1:**
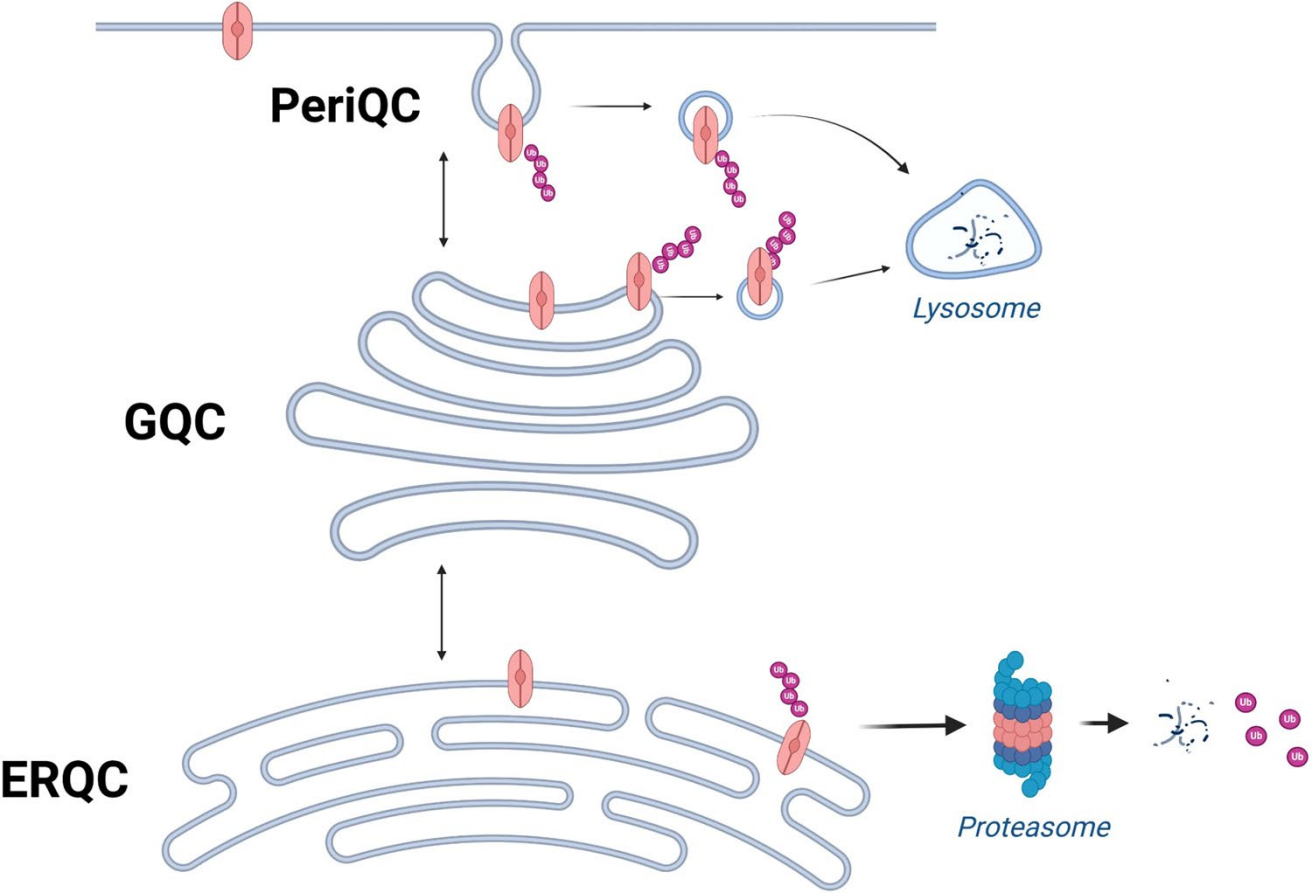
Schematic view of the CFTR QC. The protein QC systems monitor the folding and trafficking of the CFTR protein along the secretory pathway. These mechanisms include ERQC, GQC, and PeriQC. CFTR is synthesized in the endoplasmic reticulum, where it undergoes ERQC. Misfolded proteins are ubiquitinated and degraded via the proteasome. Proteins that are misfolded and escape ERQC may be recognized at the Golgi, ubiquitinated, and targeted for lysosomal degradation (GCQ). If they pass the GCQ, the proteins are trafficked to the PM. Once they are no longer required or have structural defects, they are ubiquitinated and sent to lysosomes for degradation (PeriQC). Figure created with BioRender.com

How are CFTR proteins degraded? In cells, a number of quality control (QC) mechanisms prevent improperly folded CFTR from reaching the PM. These systems have been extensively discussed in the literature [29–31]. To provide a brief overview, distinct QC systems operate along the secretory pathway including ERQC, Golgi QC (GQC), and Peripheral QC (PeriQC) (Fig. 1). Despite decades of research, however, there is still little consensus on the specific components of the different QC systems. Along this review, the readers will find several examples of drugs or drug targets whose activity on mutant CFTR was dependent on the experimental settings, with particular reference to the cell model utilized. As mentioned above, the cell background may influence the rescue of CFTR by proteostasis regulators, including drugs targeting specific components of the QC systems that, due to their redundancy, could vary in terms of expression level and biological role in different cell models [12, 27]. Thus, also studies of the QC systems, originally performed on easy-to-culture, laboratory cell models amenable to genetic manipulation, are now moving towards more complex, disease-relevant cell models, such as the immortalized bronchial CFBE41o- cell line. The technical constraints that limit the possibility of investigating the interplays between endogenous F508del-CFTR and native QC systems in primary epithelial cells will be thoroughly discussed in the last paragraph of this review.

In 1995, Kopito's groundbreaking research highlighted CFTR's rapid polyubiquitination and subsequent proteasomal degradation [5]. Notably, this study also observed that inhibiting proteasomal degradation with lactacystin, a 20S proteasome inhibitor, led to the accumulation of polyubiquitinated CFTR. Polyubiquitinated F508del-CFTR accumulates in detergent-insoluble aggregates, making it non-functional and unrecoverable, thus suggesting that blocking the proteasome is an ineffective strategy for rescuing the misfolded channel [5]. Therefore, there was soon a general consensus that action needed to be taken upstream by inhibiting channel ubiquitination *i.e.* impede channel degradation without compromising its capacity to mature and function effectively. In a pioneering study, Cyr's group demonstrated that inactivation of the Hsc70-CHIP E3 complex, which is involved in CFTR ubiquitination and degradation, resulted in increased levels of F508del-CFTR [32, 33]. By culturing cells at 27 °C, a permissive temperature for CFTR folding/maturation [34], this non-ubiquitinated form of F508del-CFTR could exit the ER and become functional at the cell surface [32]. This observation represents a pivotal moment in CFTR research, as it suggested, for the first time, the feasibility of developing drug combinations involving ubiquitination inhibitors and treatments able to correct trafficking defects to enhance the functional rescue of F508del-CFTR [32]. Targeting the ubiquitination machinery became soon one of the most promising therapeutic approaches to treat CF patients. A very recent work from Kopito’s lab confirms that preventing protein ubiquitination led to a full stabilization of the protein even if a part of the ubiquitin-mediated degradation is proteasome independent [35]. Indeed, cell surface F508del-CFTR, partially rescued by low temperatures and/or CFTR correctors, still exhibits conformational defects that lead to its internalization and subsequent elimination by ubiquitin-dependent endo-lysosomal degradation [36, 37] (see Fig. 1).

Here, we will discuss the direct targeting of ubiquitin machinery as a strategy to increase the protein amount of CFTR misfolded variants (class II). Notably, the possibility of indirectly increasing channel stability has also been investigated, for example by targeting phosphorylation [38–44], sumoylation [45], or methylation [40, 46], but this topic will not be covered by this review.

**Fig. 2:**
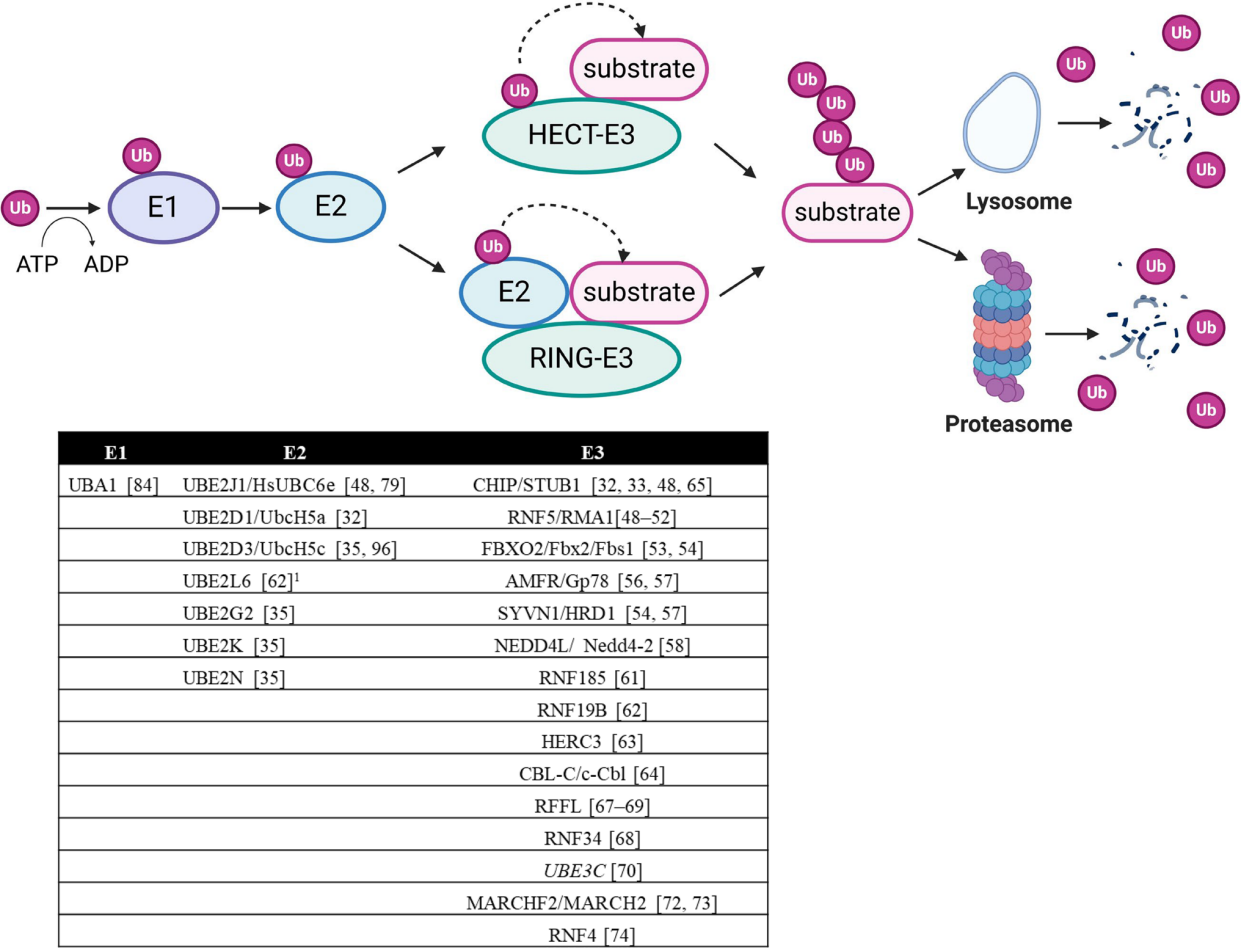
The cellular machinery involved in CFTR ubiquitination. Protein ubiquitination is controlled by E1 (ubiquitin-activating), E2 (ubiquitin-conjugating) and E3 (ubiquitin-ligating) enzymes. The table below shows which members of the ubiquitin machinery are involved in the ubiquitination and degradation of CFTR in human cells. The E3 enzyme UBE3C is in italics because there is evidence that it regulates the degradation of F508del-CFTR independently of its ubiquitin ligase activity. ^1^This is a pre-print publication not yet peer-reviewed. Figure created with BioRender.com

The ubiquitination machinery is composed of a cascade of three steps including ubiquitin activation by E1 enzymes, transfer of activated ubiquitin to E2 conjugating enzymes, and eventual ligation to the target protein by E3 ligases. In humans we have two E1, ~ 40 E2 and about 600–700 E3 [47] (Fig. 2). All three classes of proteins have been considered as potential targets to increase the stability of CFTR, and progress in the field is discussed below.

## Targeting E3: one thousand and one E3 ligases regulating CFTR?

Figure 2 and Table 1 provide a comprehensive compilation of E3 ligases participating in the ubiquitination process of CFTR, with their number continuously growing. The first breakthrough in understanding the ERQC mechanisms responsible for recognizing the misfolded F508del-CFTR protein emerged from the pioneering work of Cyr's group in 2001 on HEK293 and COS-7 transfected cells, with the identification of CHIP, also termed STUB1, as the first E3 ubiquitin ligase involved in F508del-CFTR ubiquitination and degradation [33]. It had been shown that CHIP operates in conjunction with Hsc70 to identify and target aberrant forms of CFTR for proteasomal degradation by facilitating their ubiquitination [33]. However, since the inactivation of Hsc70-CHIP was unable to completely prevent F508del-CFTR degradation, it was postulated the existence of at least a second distinct E3 ligase involved in recognizing and ubiquitinating F508del-CFTR [32]. This ligase was subsequently identified as RNF5 (also known as RMA-1) in transfected HEK293 cells [48]. The ER-anchored E3 ligase RNF5, paired with the E2 enzyme UBE2J1, was demonstrated to play a crucial role in recognizing the assembly status of both the WT- and the F508del-CFTR variant at the N-terminus detecting folding anomalies and facilitating proteasomal degradation [48]. This study demonstrated that the two ubiquitin ligases RNF5 and CHIP operate sequentially at the ER membrane and cytosol to monitor the folding status of CFTR and F508del-CFTR. Specifically, it has been shown that RNF5 can identify folding defects in F508del-CFTR while translation occurs, while CHIP appears to act post-translationally. This suggested that dual targeting of both molecular complexes would be necessary to enable F508del-CFTR to bypass QC mechanisms [48]. The targeting of RNF5 as a potential therapeutic strategy for the functional rescue of F508del-CFTR was further investigated. Notably, it has been shown that in vivo suppression of RNF5 in F508del-CFTR transgenic mice improves intestinal malabsorption while increasing CFTR activity in intestinal epithelial cells [49]. Additionally, a specific small molecule inhibitor of RNF5 (inh-2) showed efficacy in both F508del-CFTR CFBE41o- cells and primary bronchial epithelial (HBE) cells derived from CF patients homozygous for the F508del variant [50]. Unexpectedly, while the molecule alone significantly rescued F508del-CFTR in both cell models, it showed an additive effect with correctors only in CFBE41o- cells, but not in primary HBE cells [50]. These results warrant further investigation to better comprehend the compound's action. Possible explanations could be that other ligases compensate the RNF5 role in primary cells, or that secondary effects of the compound prevent an additive effect with correctors in terms of CFTR rescue. Recently, the investigation of the structure–activity relationships of inh-2 derivatives highlighted an inh-2 analog (analogue 16) as a compound endowed with a greater ability in improving the F508del-CFTR rescue induced by ELX/TEZ in CFBE41o- cells [51]. The small molecule compound FX12, an RNF5 degrader, increased mature F5408del-CFTR levels in BHK cells when combined with TEZ or LUM. However, it was ineffective in differentiated human primary airway epithelial cells homozygous for F508del-CFTR [52]. Meanwhile, in 2002, the potential involvement of the ubiquitin ligase FBXO2 (also known as Fbx2 or Fbs1) in the degradation of F508del-CFTR transfected in HEK293T cells was proposed [53]. FBXO2 exhibited a specific binding affinity to proteins bearing N-linked high-mannose oligosaccharides, subsequently contributing to their ubiquitination and degradation [53]. Most importantly, the involvement of FBXO2 with F508del-CFTR degradation was further substantiated by the subsequent research conducted by Ramachandran et al*.* in primary CF airway epithelial cells in 2016 [54]. A partial restoration of F508del-CFTR-mediated Cl^−^ transport in primary cultures of human cystic fibrosis airway epithelia was achieved through the depletion of the ubiquitin ligase FBXO2. Furthermore, the knockdown of FBXO2, in combination with the corrector compound 18, demonstrated an additional potentiation of the rescue of F508del-CFTR-mediated Cl^−^ conductance [54].

**Table 1:**
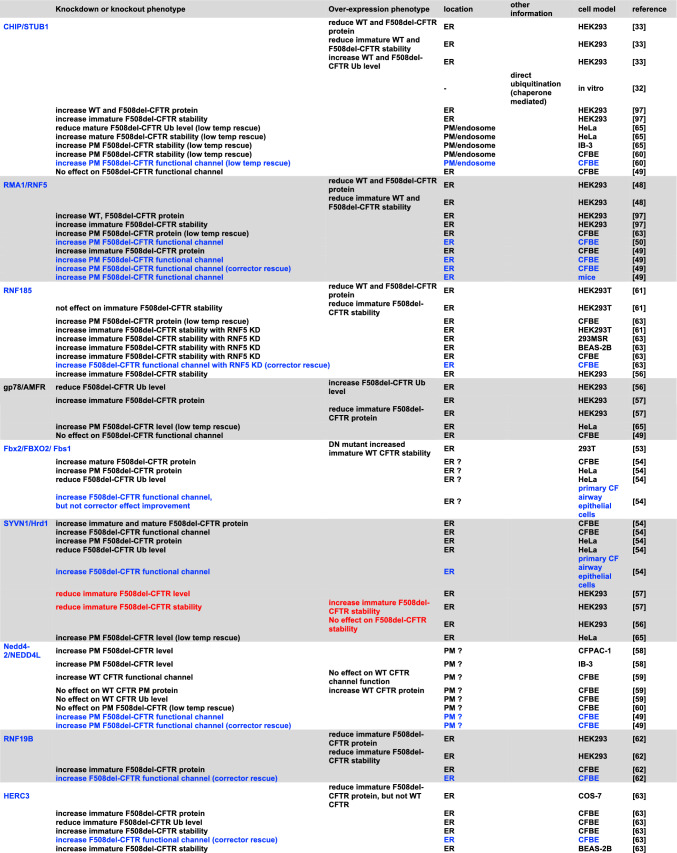

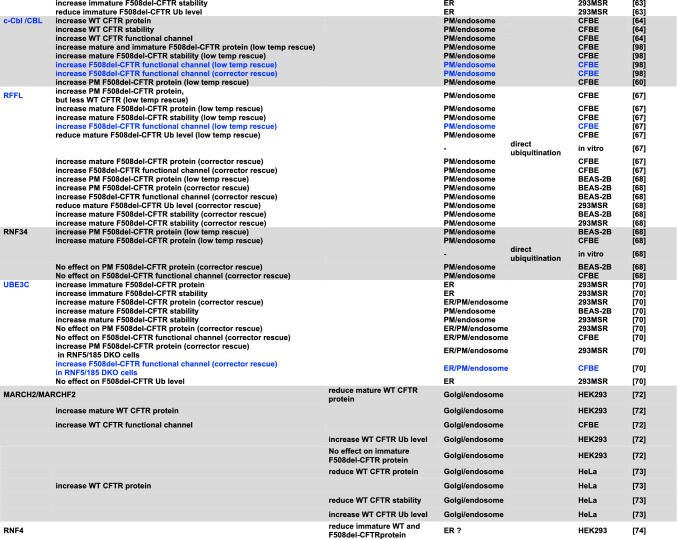
CFTR-related E3 ligases

Blue letters denote E3 ligases associated with enhanced CFTR function in an epithelial cell culture model following functional inhibition. Red letters signify the adverse effects on CFTR improvement

In 2004, Gnann et al. conducted a genetic analysis in yeast, revealing that the degradation of CFTR requires the involvement of the ubiquitin protein ligases Der3p/Hrd1p and Doa10p [55]. Morito and colleagues investigated the involvement of two mammalian proteins structurally and functionally related to yeast Hrd1p, namely AMFR (also known as gp78) and SYVN1 (also known as HRD1), in 2008 [56]. Their study demonstrated that AMFR, but not SYVN1, actively participates in ERQC of F508del-CFTR in HEK293 cells. AMFR was found to specifically promote the ubiquitylation of F508del-CFTR [56]. Furthermore, they demonstrated that AMFR acts as an E4 ligase, extending the polyubiquitin chains on F508del-CFTR initiated by RNF5 [56]. A subsequent report further confirmed the involvement of the AMFR ubiquitin ligase in F508del-CFTR ubiquitination and degradation in HEK293 cells [57]. Interestingly, although SYVN1 was not found to directly participate in controlling F508del-CFTR protein stability, it was demonstrated to play a role in the ubiquitination and subsequent degradation of AMFR [57]. In 2016, Ramachandhran et al*.*, while confirming the involvement of FBXO2 in F508del-CFTR ubiquitination in HeLa cells, also demonstrated the participation of the E3 ligase SYVN1 [54]. The authors not only presented evidence of SYVN1 involvement in F508del-CFTR proteostasis in HeLa and CFBE41o- transfected cells but also demonstrated partial restoration of F508del-CFTR-mediated Cl^−^ transport in primary cultures of human cystic fibrosis airway epithelia upon downregulation of SYVN1. Furthermore, the knockdown of SYVN1 enhanced the effect of corrector compound 18 on rescuing F508del-CFTR -mediated Cl^−^ conductance [54].

In 2009, NEDD4L (also known as Nedd4-2) was identified as another ubiquitin ligase capable of interacting with both WT- and F508del-CFTR in CFPAC-1 cells (a pancreatic adenocarcinoma cell line from a patient carrying F508del-CFTR) [58]. NEDD4L knockdown increased the PM level of functional F508del-CFTR in CFPAC-1 and IB-3 bronchial cells. The interaction between NEDD4L and F508del-CFTR was reduced by dexamethasone, which has the ability to rescue the mutated channel [58]. However, in 2012 Koeppen and colleagues showed that NEDD4L did not affect WT-CFTR Cl^−^ currents in *Xenopus* oocytes and did not ubiquitinate and regulate WT-CFTR expressed in CFBE41o- cells [59], and in 2015 Fu et al*.* showed no effect of NEDD4L knockdown on the surface stability of F508del-CFTR expressed in CFBE41o- cells [60].

In 2013, by using HEK293T cells transfected with F508del-CFTR, a new ubiquitin ligase participating in ERQC that recognizes the misfolded F508del-CFTR was identified as RNF185 [61]. RNF185 is homologous to RNF5 (more than 70% of sequence identity), and its silencing stabilizes CFTR variant proteins. Turnover analyzes indicate that, like RNF5, RNF185 targets CFTR for co-translational degradation [61].

Recently identified ubiquitin ligases involved in ERQC include RNF19B in HEK293 and CFBE41o- cells [62]^1^ and HERC3 in different transfected cell lines [63]. Notably, HERC3, which exhibits a certain degree of selectivity, functions independently of the ubiquitin ligases RNF5 and RNF185 in facilitating the ubiquitination, retrotranslocation, and degradation of F508del-CFTR [63].

Initial investigations of the ubiquitin ligases involved in the ubiquitination and degradation of F508del-CFTR at the PM as the PeriQC mechanism were carried out by Swiatecka-Urban [64] and the group led by Lukacs [65] (for a recent review on CFTR PeriQC see [29]). As previously mentioned, ubiquitinated CFTR at the PM is rapidly internalized and delivered to the lysosome for degradation [66]. Swiatecka-Urban’s group focused on studying the role of CBL-C (also known as c-Cbl) in CFTR PeriQC. Notably, their pivotal findings demonstrated the interaction between CBL-C and CFTR in primary differentiated human bronchial epithelial cells (HBE) (homozygous WT-CFTR) and F508del-CFTR in CFBE41o- cells. By using CFBE41o- cells transfected with F508del-CFTR, they proposed a dual regulatory role for CBL-C in CFTR function: firstly, by acting as an adaptor protein and facilitating CFTR endocytosis through an ubiquitin-independent mechanism, and secondly, by ubiquitinating CFTR in early endosomes, thus promoting its lysosomal degradation [64].

In 2010, Lukacs and colleagues employed a distinct approach to investigate PeriQC responsible for the degradation of misfolded membrane proteins that escape from ERQC or become damaged at PM [65]. By downregulating 33 E3 ligases involved in the downregulation of PM receptors in HeLa cells, they identified CHIP, AMFR, and SYVN1 as the E3 ligases involved. However, only CHIP downregulation demonstrated efficacy in increasing F508del-CFTR stability at the PM in IB3 bronchial cells, revealing an overlapping role between constituents of CFTR QC at the ER and the PM, with CHIP identified as the primary E3 ligase involved [65]. Given that the ablation of CHIP and CBL-C only partially inhibited the rapid elimination of F508del-CFTR from the PM [64, 65] Lukacs and colleagues sought additional E3 ligases involved in the peripheral QC of F508del-CFTR. In 2018, Lukacs's group screened 636 E3 ubiquitin ligases in CFBE41o- cells overexpressing F508del-CFTR and identified RFFL as one of the primary E3 ligases involved in chaperone-independent ubiquitination and recognition of F508del-CFTR in PeriQC. RFFL directly interacted with mature F508del-CFTR at the PM and endosomes, recognizing unfolded structures of the channel. This interaction stimulated ubiquitination, resulting in rapid endocytosis and lysosomal degradation [67]. A recent addition to the PeriQC involved in controlling F508del-CFTR stability at the PM is RNF34 [68]. RNF34 participates in the CFTR PeriQC in parallel to RFFL, directly recognizing the NBD1 domain and selectively promoting the ubiquitination of unfolded proteins. Simultaneous ablation of RNF34 and RFFL in different cell lines (BEAS-2B, CFBE41o-, 293MSR) transfected with F508del-CFTR, dramatically inhibits the degradation of mature F508del-CFTR at the PM after Trikafta treatment [68]. Recent chemical array screening identified α-tocopherol succinate (αTOS) as an RFFL ligand [69]. αTOS directly binds to the substrate-binding region of RFFL, blocking the interaction between RFFL and its substrates, such as misfolded CFTR. In airway epithelial BEAS-2B cells, αTOS modestly increases cell surface F508del-CFTR levels upon Trikafta treatment by reducing RFFL-mediated peripheral CFTR degradation [69]. However, further research is needed to evaluate αTOS's efficacy in differentiated human primary airway epithelial cells homozygous for F508del-CFTR.

Moreover, there is experimental evidence suggesting the involvement of an additional ubiquitin ligase, UBE3C, in both ERQC and PeriQC control of CFTR [70]. UBE3C played a crucial role in the ERQC of mutated CFTR through an RNF5/RNF185-independent mechanism. Downregulation of UBE3C in transfected cell lines led to an increase in the pool of F508del-CFTR, which can be corrected by Trikafta correctors. Surprisingly, UBE3C had minimal impact on the ubiquitination of immature F508del-CFTR, and expression of its catalytically inactive variant demonstrated a similar reduction in F508del-CFTR levels compared to its wild-type counterpart, suggesting that its effect is not linked to its E3 ligase activity [70]. Additionally, the downregulation of UBE3C resulted in increased PM stability of rescued F508del-CFTR and T70-CFTR, a class VI CFTR variant known for accelerated PM turnover [70]. This suggests the involvement of UBE3C in PeriQC as well.

In 2013, MARCHF2 (also known as MARCH2) was identified as a ubiquitin ligase involved in GQC that interacts with CFTR by using HEK293 cells transfected with WT-CFTR. MARCH2 is a Golgi-localized, membrane-associated ubiquitin ligase. Golgi-localized CFTR-associated ligand (CAL) and syntaxin 6 (STX6) regulate the abundance of mature, post-ER CFTR, by forming a CAL/STX6/CFTR complex (CAL complex) that promotes CFTR degradation in lysosomes of HEK293 cells [71]. The authors suggest that the recruitment of the E3 ubiquitin ligase MARCH2 to the CAL complex and subsequent ubiquitination of CFTR are responsible for the CAL-mediated lysosomal degradation of mature CFTR [72]. Knockdown of MARCH2 has been observed to elevate WT-CFTR protein levels in HeLa cells, though its impact on variant CFTR remains unexplored [73].

Finally, it should be mentioned that the SUMO-targeted ubiquitin ligase, RNF4, was found to be involved in F508del-CFTR stability in transfected HEK293 cells. Since Hsp27 promotes the SUMOylation of F508del-CFTR forming poly-SUMO chains, these poly-SUMO chains are likely recognized by the SUMO-targeted ubiquitin ligase RNF4, which may lead to F508del-CFTR polyubiquitination and proteasomal degradation [74].

## Targeting E2: the dark side of the ubiquitination machinery

About 40 ubiquitin-conjugating enzymes (E2s) involved in ubiquitin or ubiquitin-like process have been identified in humans. They are in the middle of the ubiquitination process receiving the activated ubiquitin from E1 enzymes. E3 enzymes bind both a substrate and an E2-Ub conjugate for transferring ubiquitin to the ε-amino group of a lysine in the target protein. Importantly, all E2 enzymes interact with one or more E3s. In addition, E2 enzymes may directly engage a target protein [75, 76].

E2 enzymes participating in the ubiquitination process of CFTR identified to date are shown in Fig. 2. Typically, the identification of the involved E2 enzyme occurred after the identification of an E3 ligase involved in CFTR ubiquitination and with the intention of elucidating the entire molecular mechanism.

The earliest indication of an E2 enzyme's role in CFTR stability was discovered in yeast. Within yeast cells, Ubc6p and Ubc7p, both E2 enzymes, were found to play a role in CFTR's polyubiquitination [77, 78]. The participation of UBE2J1 (also known as HsUBC6e), the mammalian counterpart of yeast Ubc6p, in the CFTR polyubiquitination has been demonstrated by Sommer and colleagues in 2002 [79]. Intriguingly, the overexpression of a dominant-negative variant of UBE2J1 enhances the stability of transfected F508del-CFTR protein in HEK293 cells [79].

Following the initial identification of the first E3 ubiquitin ligase (CHIP) [33], efforts were directed towards uncovering the entire molecular machinery responsible for F508del-CFTR degradation. As mentioned above CHIP was identified as the E3 enzyme responsible for ubiquitinating Hsc70-bound F508del-CFTR. The E2 involved in this molecular pathway was identified as the E2 enzyme UBE2D1 (also known as UbcH5a) [32]. Notably UBE2D1 belongs to a family of conserved E2 proteins, alongside UBE2D2 (also known as UbcH5b) and UBE2D3 (also known as UbcH5c), which share about 90% identity with each other [80]. Beyond UBE2D1, purified CHIP has been observed to engage with both UBE2D2 and UBE2D3, but their participation in F508del-CFTR ubiquitination was not investigated [32]. Evaluation of the impact of the overexpression of the WT and dominant-negative form of three distinct E2 enzymes (UBE2D1, UBE2J1 and UBE2G1/UBC7) in HEK293T cells overexpressing WT- or F508del- CFTR revealed the greatest effect of the UBE2D1 in modulating F508del-CFTR protein amount. Overexpression of WT-UBE2D1 led to a reduction in both CFTR band B and band C. Conversely, overexpression of the dominant-negative form increased levels of both CFTR bands increasing the half-life of the protein. Overexpression of UBE2J1 also exhibited efficacy, although its dominant-negative form did not, while UBE2G1 had no observable effects [32].

In 2010 Lukacs’ group demonstrated that the same Hsc-70-CHIP E3 participates both in the ERQC and the PeriQC. In this case, however, the authors move their attention to UBE2D3, the previously mentioned cognate E2 enzyme of CHIP. Downregulation of UBE2D3 increased the amount of F508del-CFTR at the PM in IB3 and HeLa cells [65].

The cognate E3 ubiquitin ligase that cooperates with the first E2 identified in controlling CFTR stability, UBE2J1, was identified several years later after its identification by the Cyr’s group [48]. UBE2J1 indeed does not interact with the E3 CHIP to ubiquitinate CFTR [32]. The cognate E3 was identified as the RNF5 protein: the RNF5/UBE2J1 complex recognizes the assembly status of both WT-and F508del-CFTR at the N-terminus, detecting folding anomalies and promoting proteasomal degradation. Intriguingly, UBE2J1 downregulation using RNA interference results in a nearly fourfold increase in F508del-CFTR band B amount when transfected into HEK293 cells [48].

A new E2-E3 molecular complex, UBE2L6 and RNF19B, involved in CFTR polyubiquitination and degradation has recently proposed. The amount of F508del-CFTR band B in CFBE41o- cells is downregulated when the E2 UBE2L6 is overexpressed and on the contrary upregulated when the E2 enzyme is silenced by siRNA [62].^2^

In a recent genome-wide CRISPR/Cas9 knockout (KO) screening in K562 human leukemic cells ectopically expressing F508del-CFTR to identify the molecular machinery involved in CFTR-F508del degradation, the top E2 hit was UBE2D3 [35]. The authors proposed that UBE2D3 can partner with both the E3 ligases RNF5 and RNF185, the two major E3 ligases identified in their system. However, knocking out the gene encoding the UBE2D3 had only modest impacts on F508del-CFTR levels and degradation kinetics [35], suggesting that other E2s can compensate for its function when it is knocked out. While they were unable to identify which E2 can compensate the lack of UBE2D3 using UBE2D3 KO cells, they identify UBE2K, UBE2G2 (cognate of AMFR E3 ligase [56]), and UBE2N in the RNF5 KO and/or RNF5/UBE2D3 KO screens, suggesting that multiple E2 mediate F508del-CFTR degradation [35].

## Targeting E1: a couple of enzymes controlling the entire ubiquitination process

Ubiquitin-activating enzyme (E1) catalyzes the formation of the thioester bond between the carboxyl terminus of ubiquitin and the cysteine active site of E1 in the presence of ATP. The mammalian genome contains two E1 genes, *UBA1* and *UBA6*, encoding two proteins sharing 40% identity of sequence. Although UBA1 and UBA6 proteins share a set of overlapping E2s, both E1s have their dedicated E2s. UBA1 is the predominant isoform in the protein degradation pathway. UBA6 is an unusual E1, as it activates two distinct modifiers, ubiquitin and FAT10. In 2016, Sorscher's research group started from the premise that the limited effectiveness of correctors could be attributed to the low levels of the F508del-CFTR protein caused by the misfolded structure of both its mRNA and protein [81]. Their study revealed that the use of PYR-41, an inhibitor of UBA1, substantially increased the levels of Band B in HeLa, HEK293, and CFBE41o- cells overexpressing F508del-CFTR, while leaving Band C unaffected. Most importantly, when PYR-41 was combined with the corrector C18, the functional recovery of the channel exhibited greater improvement compared to using the corrector alone. Notably, this combined approach also yielded positive outcomes for the E92K-CFTR variant [81]. These results once again underscore the significance of elevating the ER-localized CFTR levels to enhance the effects of correctors. Despite the potential value of this strategy, doubts have arisen regarding the specificity of PYR-41. This is due to evidence demonstrating that the compound exhibits comparable or even greater inhibitory effectiveness against various deubiquitinases. Additionally, PYR-41 has also been found to exert inhibitory effects on certain protein kinases [82].

In a subsequent investigation, Brodsky's team explored the impacts of several structural analogues of PYR-41 on F508del-CFTR. They singled out a compound exhibiting diminished toxicity and enhanced efficacy, effectively facilitating the rescue of F508del-CFTR induced by LUM in HEK293, FRT, and CFBE41o- cells [83].

Recently, the effect of a newly discovered highly specific UBA1 inhibitor, TAK-243 [84], on F508del-CFTR has been thoroughly investigated [84]. TAK-243 treatment of CFBE41o- cells induced a dose-dependent increase in F508del-CFTR band B while band C remained unaffected. Downregulation of UBA1 showed a similar result while downregulation of UBA6 was totally ineffective. The TAK-243 effect was demonstrated to be related to its ability to prevent F508del-CFTR ubiquitination and increase its stability. While TAK-243's augmentation of band B expression alone does not lead to functional protein rescue, combining it with the correctors ELX and TEZ (Trikafta therapy components) exhibited improved maturation of F508del-CFTR compared with only correctors treatment, indicating that the extent of channel rescue is closely tied to protein abundance. This pharmacological approach is not confined to F508del-CFTR; it could extend to other CFTR class II variants. The effects of TAK-243 were tested on eight different variants expressing defective maturation in CFBE41o- cells (L206W, R347P, S492F, M1101K, R334W, R560T, R1066C, and N1303K). When combined with ELX and TEZ correctors, TAK-243 consistently boosted channel rescue for all variants taken into account, even for variants not covered by approved Trikafta therapy, like N1303K, the second most common class II CFTR variant. Notably, the most important result is that TAK-243's effectiveness was confirmed using human primary airway epithelial cells with diverse genotypes. However, despite the promised results in CFBE41o-, not all variants responded similarly when tested in primary epithelium from patient cells. For some variants like R1066C and R347P, TAK-243 did not exhibit improvements in chloride conductance, highlighting the importance of the cellular environment in predicting compound efficacy. Positive results in cell lines might not guarantee the same efficacy in patients' cells [84]. Primary human airway epithelial cells, including human bronchial epithelial cells (HBE) and nasal epithelial cells (HNE) grown at air–liquid interface (ALI), represent the gold standard in the study of CF disease pathogenesis. These cells are instrumental in screening compounds before they advance to clinical trials and evaluating the functional response of CFTR variants to drugs [22].

## Targeting deubiquitinating enzymes: the other side of the same coin

In the regulation of protein ubiquitination, an important role is played by deubiquitinating enzymes (DUBs), which counteract the ubiquitin cascade by removing ubiquitin or polyubiquitin chains from proteins. Nearly 100 DUBs have been identified in humans, and given their pivotal role in regulating protein homeostasis, numerous studies have highlighted their involvement in diverse cellular processes and in numerous diseases. This has led to the suggestion that DUBs may represent promising therapeutic targets [85].

The first DUB responsible for the CFTR deubiquitination was identified as USP19 by Hassink and colleagues in 2009. In HEK293T transfected with F508del-CFTR, USP19 was described as an ER-localized DUB capable of rescuing F508del-CFTR from ERAD [86].

Subsequently, following an activity-based chemical screen, USP10 was identified as a CFTR deubiquitinase. In human airway epithelial cells and primary bronchial epithelial cells, USP10 has been demonstrated to interact with and deubiquitinate WT-CFTR in early endosomes, resulting in enhanced endocytic recycling of CFTR, which in turn increases the CFTR protein concentration at the PM [87, 88]. A subsequent study, however, demonstrated that the downregulation or overexpression of USP10 had no effect on the amount of F508del-CFTR protein rescued by LUM in transfected CFBE41o- cells. Instead, it identified USP13 as a DUB involved in the regulation of F508del-CFTR persistence in the PM of CFBE41o- cells treated with LUM [89].

A novel approach to stabilizing unstable proteins has been proposed, utilizing heterobifunctional stabilizers, termed DUBTACs. These are composed of a recruiter of a DUB linked to a protein-targeting ligand, which binds and deubiquitinates a protein of interest. A DUBTAC based on the use of the K48 ubiquitin-specific deubiquitinase OTUB1 was demonstrated to be effective in rescuing F508del-CFTR. The OTUB1 recruiter EN523, which covalently binds to a DUB allosteric cysteine without inhibiting DUB function, was linked to LUM to target F508del-CFTR. The treatment of CFBE41o- and primary CF bronchial epithelial cells with this DUBTAC demonstrated an increase in both protein level and functional recovery of F508del-CFTR compared to LUM alone [90].

## Targeting protein ubiquitination is still a potentially valuable therapeutic approach in Cystic Fibrosis?

The improvement of Trikafta therapy is both necessary and achievable, since it appears to not fully normalize to the WT level when applied to F508del variant [21, 91]. This is particularly crucial given the expansion of Trikafta to a substantial number of class II variants, whose effectiveness may be suboptimal and needs of enhancement [21, 22]. Additionally, it is noteworthy that Trikafta could potentially have an impact also on nonsense variants in combined therapies [92]. Improving Trikafta therapy can be achieved increasing the protein stability of CFTR variants. TEZ/ELX-rescued mature F508del-CFTR is less stable than WT-CFTR, as approximately 40% of TEZ/ELX-rescued mature F508del-CFTR was lost after a 6-h chase in CFBE41o- cells, while WT-CFTR levels remained unchanged [91]. The inclusion of IVA, another component of Trikafta, might further decrease the stability of TEZ/ELX-rescued mature F508del-CFTR. Chronic treatment with IVA has been shown to destabilize the cell surface F508del-CFTR [93].

As previously mentioned, the rapid degradation of CFTR variants occurs through ubiquitin-dependent proteasomal degradation. Kopito's groundbreaking research has already shown that proteasome inhibition is ineffective in the recovery of CFTR variants as polyubiquitinated proteins, even if they are not degraded, become insoluble in the cytosol and lose their functionality [5]. To achieve an elevated level of variants CFTR that is both foldable and capable of reaching the PM as a functional channel, it is crucial to inhibit CFTR ubiquitination, a process that facilitates retrotranslocation from the endoplasmic reticulum to the cytosol. Furthermore, the advantage of preventing ubiquitination is that it not only impedes proteasomal degradation but also inhibits ubiquitin-dependent lysosomal degradation [36].

Targeting the ubiquitination pathway could be a promising strategy to enhance the efficacy of Trikafta.

The previous findings indicate that a significant portion of Trikafta-rescued F508del-CFTR is subject to ubiquitination [91]. The ubiquitination level in TEZ/ELX-rescued F508del-CFTR was at least 1.5 times higher compared to WT-CFTR in CFBE41o- cells [91]. Additionally, F508del-CFTR remained partially ubiquitinated following Trikafta treatment, with the ubiquitination level reduced when CFTR-associated E3 ligase RFFL was knocked down [68]. Importantly, inhibiting the ubiquitin-activating enzyme (UBA1) during TEZ/ELX treatment leads to an increase in mature F508del-CFTR levels and channel function in CFBE41o- cells and differentiated human primary airway epithelial cells homozygous for F508del-CFTR [84]. Additionally, inhibition of the ubiquitin ligase RFFL improves the cell surface stability of Trikafta-rescued F508del-CFTR and enhances channel function in airway epithelial cell lines [68, 69].

Which elements of the ubiquitin machinery are the most effective targets for increasing the stability of CFTR variants? E1, E2 or E3 enzymes?

Focusing on E1, located at the initiation of the ubiquitin machinery, is theoretically the most effective intervention strategy. A highly specific inhibitor of the E1 enzyme (TAK-243) is already available and is in clinical trials for cancer treatments (*e.g.,* NCT03816319 and NCT02045095). Regarding the possible use of TAK-243 in CF patients as a pharmacological treatment, encouraging findings demonstrate its efficacy on primary airway epithelia, at least for some specific variants, suggesting its potential advancement into clinical applications [84]. However, the molecule's potential toxicity demands careful consideration due to its inhibition of the entire ubiquitin cascade. Nevertheless, it has been shown that achieving complete or nearly complete UBA1 inhibition, which could halt the entire ubiquitination process, is probably not required to achieve therapeutic effects. Interestingly, chronic administration of low doses of TAK-243 was well tolerated by CFBE41o- cell cultures and proved sufficient to enhance the effects of correctors [84]. This is consistent with the results of Brodsky’s group working on PYR-41 analogs [83].

**Fig. 3:**
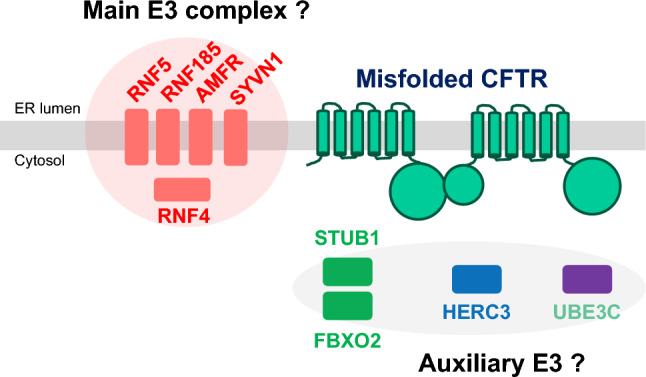
E3 ligases for CFTR ERQC. According to BioGRID, RNF5 and RNF185 could form a complex with AMFR, SYVN1, and RNF5. This complex may play a primary role in CFTR ERQC. In addition to this pathway, cytosolic E3 ligases, such as CHIP, which potentially binds to FBXO2, as well as HERC3 and UBE3C, may serve as auxiliary pathways for CFTR ERQC

**Fig. 4:**
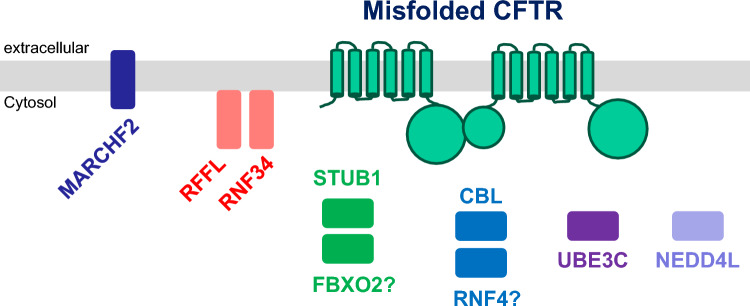
E3 ligases for CFTR PeriQC. The RFFL-RNF34 complex could play a primary role in the chaperone-independent CFTR PeriQC. The chaperone-dependent E3 ligase CHIP may form a complex with FBXO2 for CFTR PeriQC. CBL may also form a complex, although the specific role of RNF4 in CFTR PeriQC remains unclear. Furthermore, MARCHF2 may contribute to an additional pathway for CFTR PeriQC, alongside the GQC, given its involvement in endosomal trafficking (PMID: 15,689,499)

**Fig. 5:**
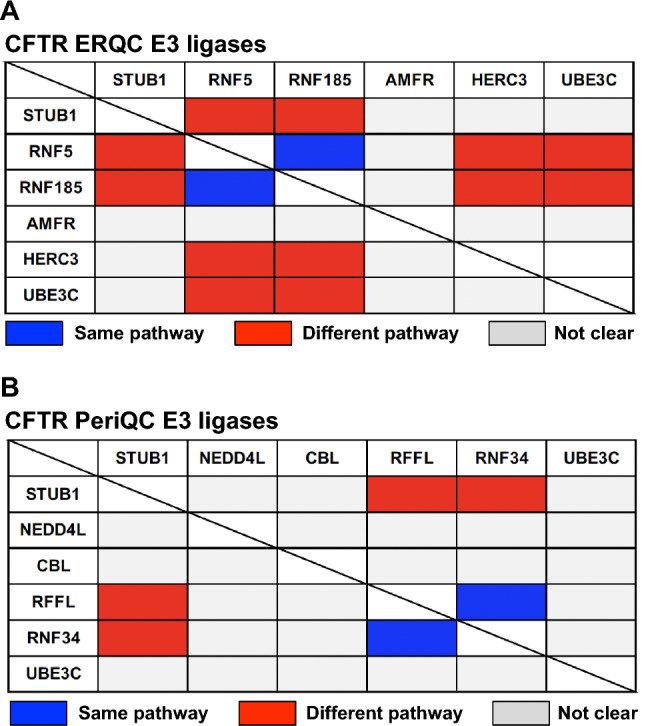
Potential functional redundancy and distinctions among CFTR-related E3 ligases. **A** Within the CFTR ERQC, RNF5 and RNF185 appear to share a redundant function [35, 61]. In contrast, cytosolic E3 ligases such as CHIP [48], HERC3 [63], and UBE3C [70] contribute additional ERAD pathways alongside the RNF5/RNF185 pathway. AMFR may operate downstream of the RNF5/RNF185 pathway [56]; however, its precise role remains unclear. **B** In the CFTR PeriQC, RFFL and RNF34 seem to share a redundant function in the chaperone-independent pathway [68]. Additionally, the chaperone-associated E3 ligase CHIP contributes to an extra PeriQC pathway. However, the functional redundancy of most E3 ligases in this context has largely remained unanalyzed

In contrast to targeting E1, targeting E3s, the last step in the ubiquitination pathway potentially allows for a more surgical pharmacological approach and minimizes disruptions to cell signaling because of the potential for more selective modulation of CFTR ubiquitination. More than 600 E3 are produced in human cells. Such a high number of enzymes may suggest a distinct specificity of each ubiquitin ligase. For this reason, the scientific community dedicated time and efforts to identifying the E3 ligases involved in CFTR ubiquitination and degradation (Fig. 3 and Fig. 4). However, over the years, several E3 ligases have been identified, each potentially playing a role in CFTR ubiquitination and degradation, as outlined in Fig. 2 and Table 1. This suggests a high degree of redundancy and possible compensatory mechanisms among E3 ligases, indicating that targeting multiple E3 ligases may be necessary for therapeutic efficacy. A recent genome-wide CRISPR/Cas9 knockout screen was used to systematically identify the most important components of the molecular machinery involved in CFTR-F508del degradation [35]. This study found that RNF5 was the top E3 ligase but surprisingly knocking it out had only modest impacts on CFTR-F508del protein levels and degradation kinetics, while preventing protein ubiquitination by the E1 inhibitor TAK243 resulted in an almost complete protein stabilization. This suggested the involvement of parallel, redundant, and/or compensatory pathways in CFTR-F508del degradation. Further analysis in RNF5 knockout cells identified RNF185 as the top E3 ligase and knocking out both RNF185 and RNF5 together had a synergistic effect on increasing reporter half-life and CFTR Band B amount. Another E3 ligase, AMFR, was found to be a weak but significant hit in the RNF5 knockout screen, suggesting its potential contribution to CFTR-F508del degradation [35]. Can we conclude that RNF5/RNF185/ AMFR are the main E3 involved in CFTR degradation? It is important to note that the experiments were performed in lymphoblast K562 cells, and the cellular context is crucial for understanding potential impacts of a treatment (see below). Recent studies have shown that, alongside the RNF5/RNF185 pathway, the HERC3 [63] and the UBE3C [70] pathways appear to play a role in determining the ERAD of CFTR-F508del. Moreover, the cytosolic chaperone-associated E3 ligase CHIP likely contributes an additional ERAD pathway, as it recognizes the cytosolic regions of misfolded CFTR [32], in contrast to RNF5/RNF185 and HERC3, both of which seem to sense the membrane-spanning domains of CFTR [63]. According to BioGRID which is a biomedical interaction repository (https://thebiogrid.org), RNF5 and RNF185 form a complex with AMFR, SYVN1, and RNF4 (Table 2). This complex may play a primary role in CFTR ERQC (Fig. 3). In addition to this pathway, cytosolic E3 ligases, such as CHIP, which potentially binds to FBXO2 (Table 2), as well as HERC3 and UBE3C, may serve as auxiliary pathways for CFTR ERQC (Fig. 3). Functional redundancy and distinctions among ERQC-associated E3 ligases have been partially unveiled through multiple knockout and knockdown experiments conducted in limited cell culture models (Fig. 5A) [63, 70]. Hence, to achieve robust CFTR stabilization at the ER, it may be necessary to concurrently inhibit multiple E3 ligases, thereby reasonably suppressing various ERAD pathways.

**Table 2:**
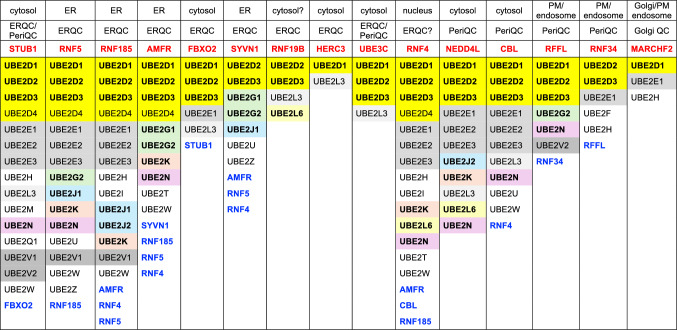
CFTR E3 associated E2 enzymes based on BioGRID

E2 enzymes binding to the E3 ligase involved in CFTR QC are depicted. Bold letters highlight those indicated to be implicated in misfolded CFTR degradation. Coloring is used to group E2 enzymes of the same family, and blue letters indicate E3 ligases binding to those involved in CFTR QC

Even following the escape from the ERQC checkpoints, variant CFTR faces potential elimination through multiple ubiquitination pathways including chaperone-mediated PeriQC mediated by CHIP [65] and chaperone-independent PeriQC mediated by RFFL [67] and RNF34 [68] (Fig. 4). Despite the fact that the detailed mechanism remains unclear, it is noteworthy that the cytosolic E3 ligase UBE3C is also involved in CFTR PeriQC [70]. RFFL and RNF34 appear to share a redundant function in the chaperone-independent pathway, alongside the chaperone-associated E3 ligase CHIP. However, the functional redundancy of most E3 ligases in the CFTR PeriQC has largely remained uncharacterized (Fig. 5B). Hence, inhibiting all E3 ligases in both the ERQC and PeriQC poses a significant challenge due to the multitude of functional E3 ligases involved in these processes.

Opting to target E2 enzymes could represent a suitable compromise between E1 and E3, as it would not halt the entire ubiquitination machinery. Given their involvement in the initial stages of the ubiquitination cascade, inhibiting E2 enzymes may prove to be more effective than E3 inhibition. Indeed, given that multiple E2 enzymes commonly associate with the E3 ligases implicated in CFTR degradation (Table 2), inhibiting selected E2 enzymes may have the potential to disrupt ubiquitination by numerous E3 ligases. It is noteworthy that the UBE2D family is involved in all CFTR-related E3 ligases, and UBE2E is additionally associated with nine E3 ligases (Table 2). Thus, targeting these E2 enzymes may be an attractive strategy to inhibit the diverse ubiquitination pathways associated with CFTR degradation in the ERQC and PeriQC. Nevertheless, as shown in Fig. 2, similar to what has been observed with E3, a substantial number of E2 enzymes are potentially implicated in the ubiquitination and degradation of CFTR variants. Hence, similar to targeting E3, focusing on a single E2 may be not adequate to achieve the desired effect, as highlighted in the above mentioned research conducted by Kopito’s group [35].

The main questions regarding the targeting of E2/E3 proteins can be summarized in two aspects: firstly, whether all identified E2/E3 ligases are relevant to CFTR stability, or if there are specific principal proteins that predominantly control the ubiquitination of mutated CFTR; secondly, whether blocking these hypothesized primary actors is effective, thus excluding the possibility that other components of the ubiquitin machinery compensate for their inactivation. The answers to these questions remain elusive, primarily because studies exploring the most pertinent E2/E3 enzymes in CFTR ubiquitination have predominantly utilized transfected immortalized non-bronchial cell lines (*e.g.* HeLa, HEK293, etc.) and bronchial cell lines (*e.g.* CFBE41o-). Notably, most of the findings obtained from these cell models have not yet been replicated in primary cells, introducing a gap in our understanding of the relevance of these enzymes in a more physiologically representative context (Table 1). The extensive utilization of immortalized cell lines is undoubtedly driven by their inherent advantages, including easy culturing, rapid expansion, and suitability for high-throughput drug screening. Additionally, the investigation of E2/E3 enzymes in CFTR degradation often relies on approaches such as gene downregulation/knockout and protein overexpression. These methods require cells that can be efficiently transfected with plasmids or RNA oligos, making immortalized cell lines a practical choice for such studies. However, the cell context is crucial as different results can be obtained according to the cellular model [84, 94]. The downregulation effect is contingent on the cell-specific expression of E2/E3 enzymes. On the other hand, E2/E3 protein overexpression could produce artifactual results since the observed effects may occur only when the protein's amount exceeds the physiological level of the enzyme. The scarcity of small-molecule inhibitors specifically designed for E2/E3 enzymes and with limited specificity [75, 95] limits the study in primary cells.

In conclusion, while challenges such as the potential toxicity associated with blocking the entire ubiquitination process (E1 targeting) and the redundancy of E2/E3 enzymes underscore the complexities of targeting the ubiquitination machinery in CF, there remains a strong rationale for further exploration. However, novel tools are required to elucidate the roles of individual E2/E3 enzymes in ubiquitinating CFTR variants in patient cells, assess their significance, and evaluate the therapeutic potential of selectively inhibiting a limited subset of these enzymes. In addition, the involvement of deubiquitinating enzymes in CFTR variants should be considered. Indeed, proof of concept for the potential application of the DUBTAC approach, which recruits a deubiquitinase to specifically enhance the stability of F508del-CFTR, has been successfully demonstrated in CFBE41o- and primary cells [90].

## Data Availability

This review article does not present any new data.

## Footnotes

1 This is a pre-print publication not yet peer-reviewed.

2 This is a pre-print publication not yet peer-reviewed.

## References

[1] Shteinberg M, Haq IJ, Polineni D, Davies JC (2021). Cystic fibrosis. Lancet.

[2] CFTR2 Variant List History | CFTR2. https://cftr2.org/mutations_history. Accessed 11 Aug 2023

[3] Veit G, Avramescu RG, Chiang AN (2016). From CFTR biology toward combinatorial pharmacotherapy: expanded classification of cystic fibrosis mutations. Mol Biol Cell.

[4] Cheng SH, Gregory RJ, Marshall J (1990). Defective intracellular transport and processing of CFTR is the molecular basis of most cystic fibrosis. Cell.

[5] Ward CL, Omura S, Kopito RR (1995). Degradation of CFTR by the ubiquitin-proteasome pathway. Cell.

[6] Jensen TJ, Loo MA, Pind S (1995). Multiple proteolytic systems, including the proteasome, contribute to CFTR processing. Cell.

[7] Van Goor F, Hadida S, Grootenhuis PDJ (2009). Rescue of CF airway epithelial cell function in vitro by a CFTR potentiator, VX-770. Proc Natl Acad Sci U S A.

[8] Accurso FJ, Rowe SM, Clancy JP (2010). Effect of VX-770 in Persons with Cystic Fibrosis and the G551D-CFTR Mutation. N Engl J Med.

[9] Bardin E, Pastor A, Semeraro M (2021). Modulators of CFTR. Updates on clinical development and future directions. Eur J Med Chem.

[10] Bobadilla JL, Macek M, Fine JP, Farrell PM (2002). Cystic fibrosis: a worldwide analysis of CFTR mutations–correlation with incidence data and application to screening. Hum Mutat.

[11] Pedemonte N, Lukacs GL, Du K (2005). Small-molecule correctors of defective DeltaF508-CFTR cellular processing identified by high-throughput screening. J Clin Invest.

[12] Li H, Pesce E, Sheppard DN (2018). Therapeutic approaches to CFTR dysfunction: From discovery to drug development. J Cyst Fibros.

[13] Farinha CM, Sousa M, Canato S (2015). Increased efficacy of VX-809 in different cellular systems results from an early stabilization effect of F508del-CFTR. Pharmacol Res Perspect.

[14] Fiedorczuk K, Chen J (2022). Mechanism of CFTR correction by type I folding correctors. Cell.

[15] Fiedorczuk K, Chen J (2022). Molecular structures reveal synergistic rescue of Δ508 CFTR by Trikafta modulators. Science.

[16] Wainwright CE, Elborn JS, Ramsey BW (2015). Lumacaftor-Ivacaftor in patients with cystic fibrosis homozygous for Phe508del CFTR. N Engl J Med.

[17] Roda J, Pinto-Silva C, Silva IAI (2022). New drugs in cystic fibrosis: what has changed in the last decade?. Therap Adv Chronic Dis.

[18] Sagel SD, Khan U, Heltshe SL (2021). Clinical Effectiveness of Lumacaftor/Ivacaftor in Patients with Cystic Fibrosis Homozygous for F508del-CFTR. A Clinical Trial Annals ATS.

[19] Lopes-Pacheco M (2019). CFTR Modulators: the changing face of cystic fibrosis in the era of precision medicine. Front Pharmacol.

[20] Middleton PG, Mall MA, Dřevínek P (2019). Elexacaftor–Tezacaftor–Ivacaftor for Cystic Fibrosis with a Single Phe508del Allele. N Engl J Med.

[21] Taylor-Cousar JL, Robinson PD, Shteinberg M, Downey DG (2023). CFTR modulator therapy: transforming the landscape of clinical care in cystic fibrosis. Lancet.

[22] Dreano E, Burgel PR, Hatton A (2023). Theratyping cystic fibrosis patients to guide elexacaftor/tezacaftor/ivacaftor out-of-label prescription. Eur Respir J.

[23] Veit G, Vaccarin C, Lukacs GL (2021). Elexacaftor co-potentiates the activity of F508del and gating mutants of CFTR. J Cyst Fibros.

[24] Tewkesbury DH, Robey RC, Barry PJ (2021). Progress in precision medicine in cystic fibrosis: a focus on CFTR modulator therapy. Breathe (Sheff).

[25] Ben Saad A, Vauthier V, Tóth Á (2021). Effect of CFTR correctors on the traffic and the function of intracellularly retained ABCB4 variants. Liver Int.

[26] Scano M, Benetollo A, Nogara L (2022). CFTR corrector C17 is effective in muscular dystrophy, in vivo proof of concept in LGMDR3. Hum Mol Genet.

[27] Pedemonte N, Tomati V, Sondo E, Galietta LJV (2010). Influence of cell background on pharmacological rescue of mutant CFTR. Am J Physiol Cell Physiol.

[28] Brusa I, Sondo E, Falchi F (2022). Proteostasis Regulators in Cystic Fibrosis: Current Development and Future Perspectives. J Med Chem.

[29] Taniguchi S, Fukuda R, Okiyoneda T (2023). The multiple ubiquitination mechanisms in CFTR peripheral quality control. Biochem Soc Trans.

[30] Amaral MD (2004). CFTR and chaperones: processing and degradation. J Mol Neurosci.

[31] Amaral MD, Hutt DM, Tomati V (2020). CFTR processing, trafficking and interactions. J Cyst Fibros.

[32] Younger JM, Ren H-Y, Chen L (2004). A foldable CFTR{Delta}F508 biogenic intermediate accumulates upon inhibition of the Hsc70-CHIP E3 ubiquitin ligase. J Cell Biol.

[33] Meacham GC, Patterson C, Zhang W (2001). The Hsc70 co-chaperone CHIP targets immature CFTR for proteasomal degradation. Nat Cell Biol.

[34] Denning GM, Anderson MP, Amara JF (1992). Processing of mutant cystic fibrosis transmembrane conductance regulator is temperature-sensitive. Nature.

[35] Riepe C, Wąchalska M, Deol KK (2023). Small molecule correctors divert CFTR-F508del from ERAD by stabilizing sequential folding states. Mol Biol Cell.

[36] Sharma M, Pampinella F, Nemes C (2004). Misfolding diverts CFTR from recycling to degradation : quality control at early endosomes. J Cell Biol.

[37] Heda GD, Tanwani M, Marino CR (2001). The ΔF508 mutation shortens the biochemical half-life of plasma membrane CFTR in polarized epithelial cells. Am J Physiol Cell Physiol.

[38] D’Amore C, Borgo C, Bosello-Travain V (2020). Diphering the role of protein kinase CK2 in the maturation/stability of F508del-CFTR. Biochim Biophys Acta Mol Basis Dis.

[39] Dahan D, Evagelidis A, Hanrahan JW (2001). Regulation of the CFTR channel by phosphorylation. Pflugers Arch.

[40] Pankow S, Bamberger C, Yates JR (2019). A posttranslational modification code for CFTR maturation is altered in cystic fibrosis. Sci Signal.

[41] Perkins LA, Fisher GW, Naganbabu M (2018). High-Content Surface and Total Expression siRNA Kinase Library Screen with VX-809 Treatment Reveals Kinase Targets that Enhance F508del-CFTR Rescue. Mol Pharmaceutics.

[42] Amaral MD, Farinha CM (2013). Post-translational modifications of CFTR: insight into protein trafficking and cystic fibrosis disease. FEBS J.

[43] Degrugillier F, Aissat A, Prulière-Escabasse V (2020). Phosphorylation of the Chaperone-Like HspB5 Rescues Trafficking and Function of F508del-CFTR. Int J Mol Sci.

[44] Liang X, Da Paula AC, Bozóky Z (2012). Phosphorylation-dependent 14–3-3 protein interactions regulate CFTR biogenesis. MBoC.

[45] Ahner A, Frizzell RA (2015). SUMOylation Modulates CFTR Biogenesis: Is the Pathway Druggable?. Curr Drug Targets.

[46] D’Amore C, Borgo C, Bosello Travain V, Salvi M (2022). KDM2A and KDM3B as Potential Targets for the Rescue of F508del-CFTR. Int J Mol Sci.

[47] George AJ, Hoffiz YC, Charles AJ (2018). A Comprehensive Atlas of E3 Ubiquitin Ligase Mutations in Neurological Disorders. Front Genet.

[48] Younger JM, Chen L, Ren H-Y (2006). Sequential quality-control checkpoints triage misfolded cystic fibrosis transmembrane conductance regulator. Cell.

[49] Tomati V, Sondo E, Armirotti A (2015). Genetic Inhibition Of The Ubiquitin Ligase Rnf5 Attenuates Phenotypes Associated To F508del Cystic Fibrosis Mutation. Sci Rep.

[50] Sondo E, Falchi F, Caci E (2018). Pharmacological Inhibition of the Ubiquitin Ligase RNF5 Rescues F508del-CFTR in Cystic Fibrosis Airway Epithelia. Cell Chem Biol.

[51] Brusa I, Sondo E, Pesce E (2023). Innovative Strategy toward Mutant CFTR Rescue in Cystic Fibrosis: Design and Synthesis of Thiadiazole Inhibitors of the E3 Ligase RNF5. J Med Chem.

[52] Ruan J, Liang D, Yan W (2022). A small-molecule inhibitor and degrader of the RNF5 ubiquitin ligase. Mol Biol Cell.

[53] Yoshida Y, Chiba T, Tokunaga F (2002). E3 ubiquitin ligase that recognizes sugar chains. Nature.

[54] Ramachandran S, Osterhaus SR, Parekh KR (2016). SYVN1, NEDD8, and FBXO2 Proteins Regulate ΔF508 Cystic Fibrosis Transmembrane Conductance Regulator (CFTR) Ubiquitin-mediated Proteasomal Degradation. J Biol Chem.

[55] Gnann A, Riordan JR, Wolf DH (2004). Cystic fibrosis transmembrane conductance regulator degradation depends on the lectins Htm1p/EDEM and the Cdc48 protein complex in yeast. Mol Biol Cell.

[56] Morito D, Hirao K, Oda Y (2008). Gp78 cooperates with RMA1 in endoplasmic reticulum-associated degradation of CFTRΔF508. Mol Biol Cell.

[57] Ballar P, Ors AU, Yang H, Fang S (2010). Differential regulation of CFTRDeltaF508 degradation by ubiquitin ligases gp78 and Hrd1. Int J Biochem Cell Biol.

[58] Caohuy H, Jozwik C, Pollard HB (2009). Rescue of DeltaF508-CFTR by the SGK1/Nedd4-2 signaling pathway. J Biol Chem.

[59] Koeppen K, Chapline C, Sato JD, Stanton BA (2012). Nedd4-2 does not regulate wt-CFTR in human airway epithelial cells. Am J Physiol Lung Cell Mol Physiol.

[60] Fu L, Rab A, Tang L (2015). ΔF508 CFTR Surface Stability Is Regulated by DAB2 and CHIP-Mediated Ubiquitination in Post-Endocytic Compartments. PLoS ONE.

[61] El Khouri E, Le Pavec G, Toledano MB, Delaunay-Moisan A (2013). RNF185 Is a Novel E3 Ligase of Endoplasmic Reticulum-associated Degradation (ERAD) That Targets Cystic Fibrosis Transmembrane Conductance Regulator (CFTR)*. J Biol Chem.

[62] Research Square (2019) Degradation of CFTR-F508del By the Ubiquitin E2 Conjugating Enzyme UBE 2L6 and the E3 Ligase RNF19B. https://www.researchsquare.com. Accessed 12 Apr 2021

[63] Kamada Y, Ohnishi Y, Nakashima C (2023). HERC3 E3 ligase provides an ERAD branch eliminating select membrane proteins. Biorxiv.

[64] Ye S, Cihil K, Stolz DB (2010). c-Cbl facilitates endocytosis and lysosomal degradation of cystic fibrosis transmembrane conductance regulator in human airway epithelial cells. J Biol Chem.

[65] Okiyoneda T, Barrière H, Bagdány M (2010). Peripheral Protein Quality Control Removes Unfolded CFTR from the Plasma Membrane. Science.

[66] Fukuda R, Okiyoneda T (2018). Peripheral Protein Quality Control as a Novel Drug Target for CFTR Stabilizer. Front Pharmacol.

[67] Okiyoneda T, Veit G, Sakai R (2018). Chaperone-Independent Peripheral Quality Control of CFTR by RFFL E3 Ligase. Dev Cell.

[68] Taniguchi S, Ito Y, Kiritani H (2022). The Ubiquitin Ligase RNF34 Participates in the Peripheral Quality Control of CFTR (RNF34 Role in CFTR PeriQC). Front Mol Biosci.

[69] Taniguchi S, Ono Y, Doi Y (2023). Identification of α-Tocopherol succinate as an RFFL-substrate interaction inhibitor inducing peripheral CFTR stabilization and apoptosis. Biochem Pharmacol.

[70] Kamada Y, Tateishi H, Nakayamada U (2023). UBE3C Facilitates the ER-Associated and Peripheral Degradation of Misfolded CFTR. Cells.

[71] Cheng J, Cebotaru V, Cebotaru L, Guggino WB (2010). Syntaxin 6 and CAL mediate the degradation of the cystic fibrosis transmembrane conductance regulator. Mol Biol Cell.

[72] Cheng J, Guggino W (2013). Ubiquitination and degradation of CFTR by the E3 ubiquitin ligase MARCH2 through its association with adaptor proteins CAL and STX6. PLoS ONE.

[73] Xia D, Qu L, Li G (2016). MARCH2 regulates autophagy by promoting CFTR ubiquitination and degradation and PIK3CA-AKT-MTOR signaling. Autophagy.

[74] Ahner A, Gong X, Schmidt BZ (2013). Small heat shock proteins target mutant cystic fibrosis transmembrane conductance regulator for degradation via a small ubiquitin-like modifier-dependent pathway. Mol Biol Cell.

[75] Stewart MD, Ritterhoff T, Klevit RE, Brzovic PS (2016). E2 enzymes: more than just middle men. Cell Res.

[76] Middleton AJ, Day CL (2023). From seeds to trees: how E2 enzymes grow ubiquitin chains. Biochem Soc Trans.

[77] Kiser GL, Gentzsch M, Kloser AK (2001). Expression and Degradation of the Cystic Fibrosis Transmembrane Conductance Regulator in Saccharomyces cerevisiae. Arch Biochem Biophys.

[78] Zhang Y, Nijbroek G, Sullivan ML (2001). Hsp70 Molecular Chaperone Facilitates Endoplasmic Reticulum-associated Protein Degradation of Cystic Fibrosis Transmembrane Conductance Regulator in Yeast. MBoC.

[79] Lenk U, Yu H, Walter J (2002). A role for mammalian Ubc6 homologues in ER-associated protein degradation. J Cell Sci.

[80] Jensen JP, Bates PW, Yang M (1995). Identification of a family of closely related human ubiquitin conjugating enzymes. J Biol Chem.

[81] Chung WJ, Goeckeler-Fried JL, Havasi V (2016). Increasing the Endoplasmic Reticulum Pool of the F508del Allele of the Cystic Fibrosis Transmembrane Conductance Regulator Leads to Greater Folding Correction by Small Molecule Therapeutics. PLoS ONE.

[82] Kapuria V, Peterson LF, Showalter HDH (2011). Protein cross-linking as a novel mechanism of action of a ubiquitin-activating enzyme inhibitor with anti-tumor activity. Biochem Pharmacol.

[83] Goeckeler-Fried JL, Aldrin Denny R, Joshi D (2021). Improved correction of F508del-CFTR biogenesis with a folding facilitator and an inhibitor of protein ubiquitination. Bioorg Med Chem Lett.

[84] Borgo C, D’Amore C, Capurro V (2022). Targeting the E1 ubiquitin-activating enzyme (UBA1) improves elexacaftor/tezacaftor/ivacaftor efficacy towards F508del and rare misfolded CFTR mutants. Cell Mol Life Sci.

[85] He M, Zhou Z, Shah AA (2016). The emerging role of deubiquitinating enzymes in genomic integrity, diseases, and therapeutics. Cell Biosci.

[86] Hassink GC, Zhao B, Sompallae R (2009). The ER-resident ubiquitin-specific protease 19 participates in the UPR and rescues ERAD substrates. EMBO Rep.

[87] Bomberger JM, Barnaby RL, Stanton BA (2009). The deubiquitinating enzyme USP10 regulates the post-endocytic sorting of cystic fibrosis transmembrane conductance regulator in airway epithelial cells. J Biol Chem.

[88] Bomberger JM, Barnaby RL, Stanton BA (2010). The deubiquitinating enzyme USP10 regulates the endocytic recycling of CFTR in airway epithelial cells. Channels (Austin).

[89] Pesce E, Sondo E, Ferrera L (2018). The Autophagy Inhibitor Spautin-1 Antagonizes Rescue of Mutant CFTR Through an Autophagy-Independent and USP13-Mediated Mechanism. Front Pharmacol.

[90] Henning NJ, Boike L, Spradlin JN (2022). Deubiquitinase-targeting chimeras for targeted protein stabilization. Nat Chem Biol.

[91] Capurro V, Tomati V, Sondo E (2021). Partial Rescue of F508del-CFTR Stability and Trafficking Defects by Double Corrector Treatment. Int J Mol Sci.

[92] Venturini A, Borrelli A, Musante I (2021). Comprehensive Analysis of Combinatorial Pharmacological Treatments to Correct Nonsense Mutations in the CFTR Gene. Int J Mol Sci.

[93] Veit G, Avramescu RG, Perdomo D (2014). Some gating potentiators, including VX-770, diminish ΔF508-CFTR functional expression. Sci Transl Med.

[94] Tomati V, Costa S, Capurro V (2023). Rescue by elexacaftor-tezacaftor-ivacaftor of the G1244E cystic fibrosis mutation’s stability and gating defects are dependent on cell background. J Cyst Fibros.

[95] Galdeano C (2017). Drugging the undruggable: targeting challenging E3 ligases for personalized medicine. Future Med Chem.

[96] Grove DE, Rosser MFN, Ren HY (2009). Mechanisms for rescue of correctable folding defects in CFTRDelta F508. Mol Biol Cell.

[97] Cihil KM, Zimnik A, Swiatecka-Urban A (2013). c-Cbl reduces stability of rescued ∆F508-CFTR in human airway epithelial cells: Implications for cystic fibrosis treatment. Commun Integr Biol.

